# Education for Pediatric Gastroenterology Pathology Reports Increases Understanding Ahead of 21st Century Cures Act Rollout

**DOI:** 10.1097/PG9.0000000000000197

**Published:** 2022-03-31

**Authors:** Leland Dunwoodie, Annette Vannilam

**Affiliations:** From the * Vanderbilt University School of Medicine, Nashville, TN; † Department of Pediatrics, Division of Pediatric Gastroenterology, Hepatology, and Nutrition, Vanderbilt University Medical Center, Nashville, TN.

## Abstract

As a result of the 21st Century Cures Act, Pediatric Gastroenterology (Peds GI) patients and caregivers are receiving pathology reports directly on their online patient portal. This quality improvement project attempted to validate an educational handout about Peds GI pathology reports and identify areas for caregiver education.

**Methods:**

Caregivers (n = 59) of patients undergoing Peds GI procedures were surveyed about their knowledge of pathology reports. Caregivers’ responses to each of 4 questions were assessed before and after reading an educational handout. These 4 questions questioned the contents and creators of pathology reports, the role of pathologists, and caregiver confidence in interpreting pathology reports.

**Results:**

Nearly one-third of caregivers did not know the role of pathologists before education, and one-fifth of caregivers did not know the contents of a pathology report. Caregivers with less than a college degree (n = 21, *P* = 0.0089), and caregivers of patients undergoing their first procedure (n = 27, *P* = 0.0022) showed a significant improvement in responses after the educational handout.

**Conclusions:**

Educational handouts can increase caregiver understanding of pathology reports, especially for those with lower education levels or those with children undergoing their first procedure.

**What is Known**

- The 21st Century Cures Act enables patients to see many test results, including pathology reports, in real time.
- Little patient education exists for Pediatric Gastroenterology pathology reports generated by upper endoscopies and colonoscopies.

**What is New**

- Caregiver education can increase understanding of pathology reports for Pediatric Gastroenterology procedures
- Caregivers (parents/guardians) of Pediatric Gastroenterology patients who have lower education levels or of patients undergoing their first Pediatric Gastroenterology procedure derive the greatest benefit from educational materials.

## INTRODUCTION

Many patient education initiatives have been utilized in the field of gastroenterology (GI). With adult GI patients, a meta-analysis of 9 studies concluded that patient education increased the likelihood of adequate bowel prep in adults (1). Furthermore, with pediatric patients, phone interactions with patients or their parents the day before the procedure were associated with increased prep compliance (2). More broadly, online patient education is associated with higher patient satisfaction during clinic visits (3). Individualizing online patient education to specific patient needs is also related to patient satisfaction (4).

Context is important to this project. At our institution, as of April 2021, pathology reports from Peds GI procedures, along with other patient data, are uploaded to the online patient portal in real time according to the 21st Century Cures Act (5). As such, this project’s specific aims included: (1) to identify opportunities and target populations for pathology report education; and (2) to develop and validate an educational handout concerning pathology reports. Patient and caregiver education can increase patient satisfaction and patient understanding as well as decrease work for providers.

## METHODS

A caregiver survey (Supplemental Digital Content File 1, http://links.lww.com/PG9/A80) and an educational handout (Supplemental Digital Content File 2, http://links.lww.com/PG9/A81) were developed in collaboration with Peds GI physicians at our institution. These materials were given to the caregivers accompanying Peds GI patients to their procedure. Survey responses before and after reading the handout were recorded on the day of the procedure.

The survey was given to adult caregivers of Peds GI patients in the holding area before Peds GI procedures. The survey was given to English-speaking caregivers who had access to the patient’s online patient portal. Caregivers who did not use the online patient portal were not given the survey.

Caregivers were given the survey and handout once their verbal consent was obtained. Caregivers were not allowed to keep either the survey or educational handout. Data were entered and stored in REDCap (Research Electronic Data Capture), which is a secure, web-based software platform designed to support data capture for research studies (6,7). Caregivers answered questions about their child’s procedure and their own educational background. Caregivers also were given the option to suggest improvements for the online patient portal.

Caregivers were asked 4 questions about pathologists/pathology reports. Three were multiple-choice questions and one was a Likert-scale-style question.

(1) “What does a pathology report contain?”

- Images collected during the procedure
- Information about the quality of the doctor who performed the procedure
- Analysis of tissue collected during the procedure (desired response)
- None of the above
- Unsure

(2) “I know what sorts of information to expect from the pathology report the patient will receive after their pediatric gastroenterology procedure.”

- Strongly disagree
- Disagree
- Neutral (desired response)
- Agree (desired response)
- Strongly agree (desired response)

(3) “Who will write the pathology report created after the patient’s pediatric gastroenterology procedure?”

- A pathologist (desired response)
- The physician who performed the procedure

(4) “Are pathologists practicing physicians? Practicing physicians graduated from medical school and then did additional training in their field of interest.”

- Yes, pathologists are practicing physicians (desired response)
- No, pathologists are not practicing physicians

After completing these 4 questions, caregivers read the educational handout. After reading the handout, caregivers were asked the same 4 questions again. Responses with >1 multiple-choice question omitted were not considered. The percent of respondents giving the desired response pre- versus posteducation were compared with a Fisher exact test (α < 0.05). Responses to the question “Are pathologists practicing physicians?” were stratified by 2 variables, educational background and the patient having had a previous pediatric gastroenterology procedure. These 2 variables are further outlined later. These responses were compared pre- and posteducation with a Fisher exact test (α < 0.05).

(a) Responses to “Are pathologists practicing physicians?” were compared for 3 groups of caregivers based upon the highest educational level they had achieved:
  (1) Some high school, high school degree, or some college
  (2) College degree
  (3) Graduate/professional degree
(b) Responses to “Are pathologists practicing physicians?” were compared for 2 groups of caregivers based upon the patient’s pediatric gastroenterology procedure history on the day of survey completion:
  (1) This is the patient’s first pediatric gastroenterology procedure
  (2) This is not the patient’s first pediatric gastroenterology procedure

Post hoc power calculations were performed. These power calculations evaluated changes in understanding before and after education for caregivers with lower educational backgrounds and those accompanying patients receiving their first procedure.

## RESULTS

Sixty caregivers of patients receiving Peds GI procedures agreed to complete the survey. One respondent omitted >1 question and was excluded, with 59 respondents included in the final data analysis.

### Caregiver’s Educational Level

**FIGURE 1.**
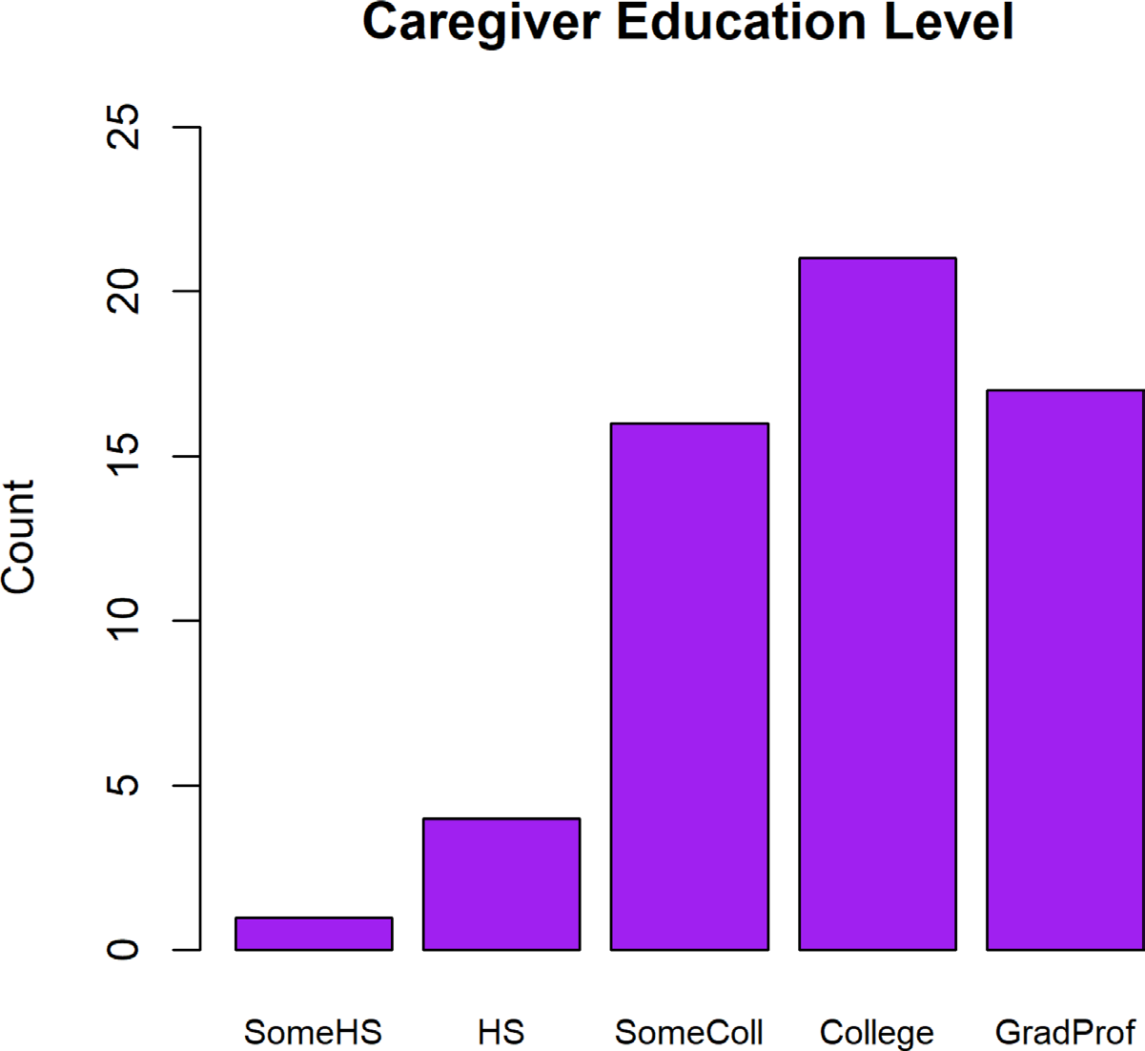
Highest educational level reached by caregiver completing survey. SomeHS = some high school; HS = high school degree; SomeColl = some college; College = college degree; GradProf = graduate or professional degree.

Of the 59 caregivers surveyed, 1.7% responded that the highest educational level they had achieved was “Some High School”; 6.8% responded “High School degree,” 27.1% responded “Some College,” 35.6% responded “College degree,” and 28.8% responded “Graduate or Professional degree” (Fig. 1).

### Child’s Procedure

**FIGURE 2.**
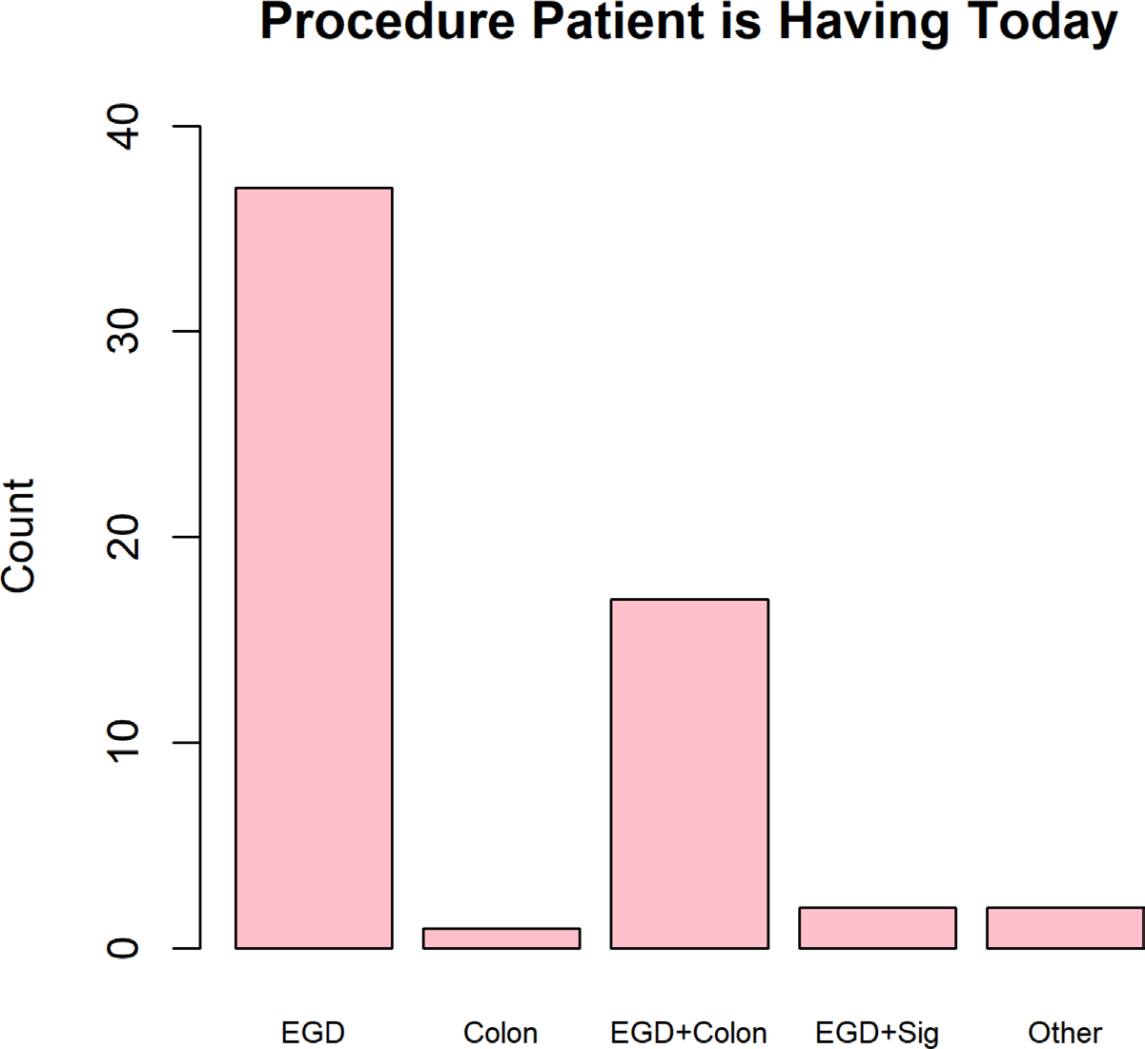
Procedure experienced by the patient with the caregiver completing the survey. “Other” includes anorectal manometry and rectal suction biopsy. EGD = esophagogastroduodenoscopy.

Of the 59 caregivers surveyed, 62.7% responded that the patient was having an esophagogastroduodenoscopy (EGD), 1.7% indicated colonoscopy, 28.8% indicated EGD + colonoscopy, 3.4% indicated EGD + flexible sigmoidoscopy, and 3.4% indicated “Other.” “Other” included anorectal manometry and rectal suction biopsy. In addition, 45.8% responded that it was the patient’s first pediatric gastroenterology procedure (Fig. 2).

### Responses Before and After reading Educational Handout

**FIGURE 3.**
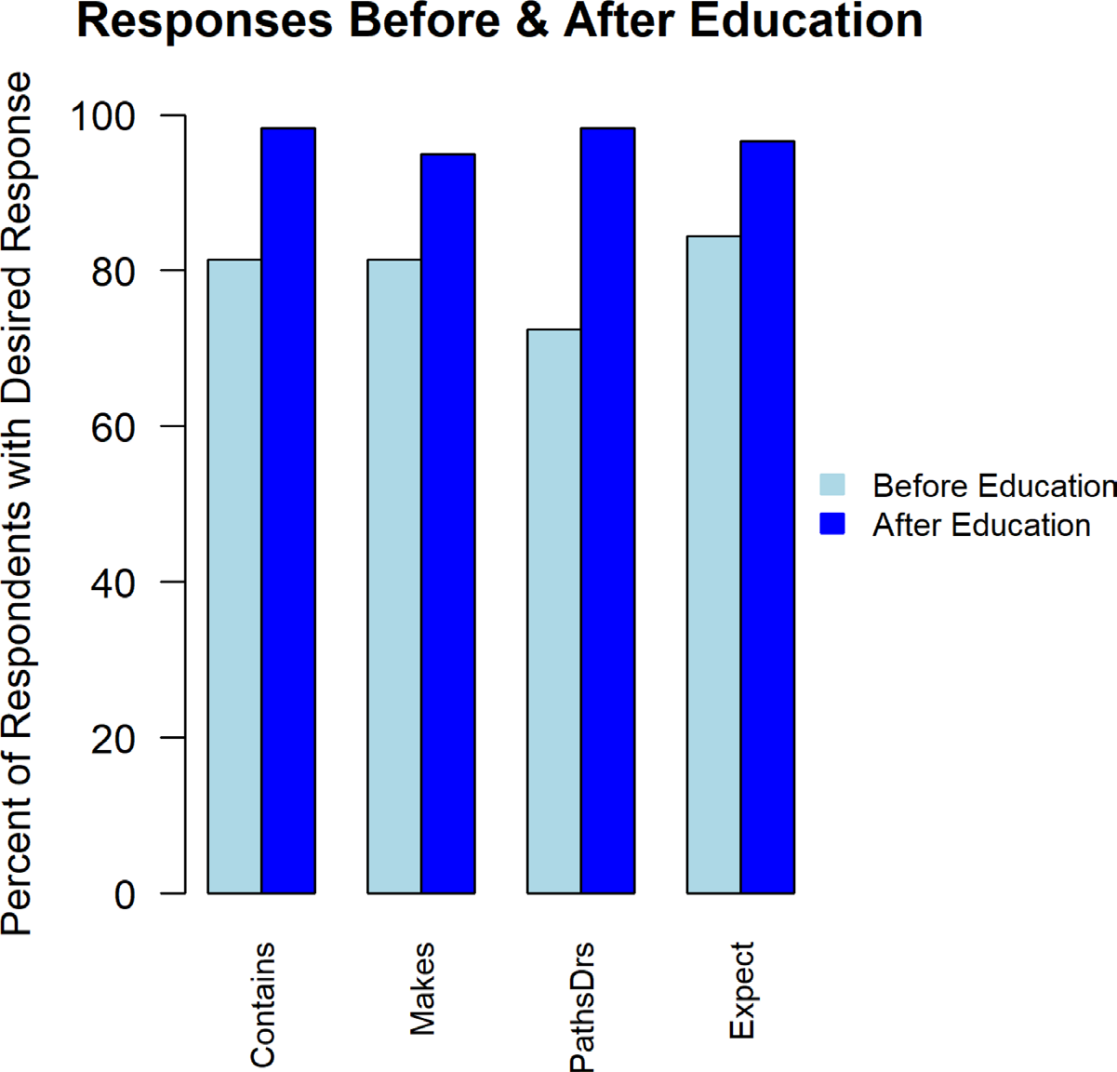
Caregiver responses to questions about pathology reports before and after reading educational handout. Contains = “After their upcoming pediatric gastroenterology procedure, the patient will receive a PATHOLOGY REPORT on MyHealthAtVanderbilt. What does a pathology report contain?” Desired response was “Analysis of tissue collected during the procedure.” Expect = “Please rate your agreement with the following statement: I know what sorts of information to expect from the pathology report the patient will receive after their pediatric gastroenterology, procedure.” Desired response was “Neutral,” “Agree,” or “Strongly Agree” as opposed to “Strongly Disagree” or “Disagree,” Makes = “Who will write the pathology report created after the patient’s pediatric gastroenterology procedure?” Desired response was “A pathologist,” PathDrs = “Are pathologists practicing physicians? Practicing physicians graduated from medical school and then did additional training in their field of interest.” Desired response was “Yes.”

For each of the 4 pathology report-related questions, the percent of respondents giving the desired response increased after reading the educational handout (question 1%–98.3% from 81.4%; question 2%–96.6% from 84.4%; question 3%–94.9% from 81.4%; question 4%–98.3% from 72.4%) (Fig. 3).

### Satisfaction With Patient Portal

One hundred percent of respondents indicated they were satisfied with the MyHealthAtVanderbilt patient portal (data not shown). Specific free-text comments offering suggestions for portal improvement were discussed with our pediatric gastroenterology faculty.

### Change in Response as a Function of Caregiver Educational Level and First Procedure Status

A significant difference (*P* = 0.0089) in the number of respondents giving the desired response to “Are pathologists practicing physicians?” was found pre- versus posteducation for caregivers with less than a college degree (n = 21). A post hoc power calculation was done treating the 21 respondents with lower educational backgrounds before and after education as 2 different groups. The change from 57% to 95% correct after education had a power of 84.9% (α < 0.05). No significant difference was found pre- versus posteducation for caregivers with a college degree (*P* = 0.1069, n = 21) or for those with a graduate or professional degree (*P* = 0.1026, n = 17).

Based upon caregiver educational background, caregiver responses to “Are pathologists practicing physicians?” pre- and posteducation compared with a Fisher exact test (Table 1).

**TABLE 1.** Caregiver responses to “Are Pathologists Medical Doctors?” stratified by caregiver education level

Caregiver education level	Yes—Are pathologists medical doctors?		
	Before handout, n (%)	After handout, n (%)	Before vs. after, *P*
Less than college degree (21)	57	95	0.0089
College degree (21)	81	100	0.1069
Graduate/professional degree (17)	81	100	0.1026

A significant difference (*P* = 0.0022) in the number of respondents giving the desired response to “Are pathologists practicing physicians?” was found pre- versus posteducation for caregivers with patients having their first Peds GI procedure (n = 27) but not for caregivers with patients who had at least one Peds GI procedure previously (*P* = 0.1042, n = 32). A post hoc power calculation was done treating the 27 respondents accompanying patients receiving their first procedure before and after education as 2 different groups. The change from 59% to 96% correct after education had a power of 92.6% (α < 0.05).

Caregiver responses to “Are pathologists practicing physicians?” for caregivers with patients having their first versus not their first Peds GI procedure (Table 2).

**TABLE 2.** Caregiver responses to “Are Pathologists Medical Doctors?” for caregivers accompanying a child to their first or not their first pediatric gastroenterology procedure

Patient’s first Peds GI procedure?	Yes—Are pathologists medical doctors?		
	Before handout, n (%)	After handout, n (%)	Before vs. after, *P*
First (27)	59	96	0.0022
Not first (32)	84	97	0.1042

## DISCUSSION

Data from this quality improvement project suggest education increases caregiver understanding of Peds GI pathology reports and pathologists’ roles. This is important with the 21st Century Cures Act making pathology reports available in real time to patients and caregivers. Caregivers should be equipped with the educational resources they need to better understand pathology reports. Pathology report education could also help patients answer common questions without needing to consult their physician.

This project has several ethical considerations. First, having caregivers complete the survey in the preoperative holding or waiting area may have decreased caregiver time spent with the patient. However, the survey only took 5–10 minutes to complete and patient wait time was often 1–2 hours. Next, administering the survey before the procedure could have created a power differential. The survey was introduced as completely optional. Still, in the preoperative holding area, caregivers meet several nurses and doctors who give them forms, such as consent forms, that must be completed before the procedure. This optional survey could have been perceived as another mandatory item that is part of the procedure process. If a power differential was created, caregivers may have felt forced to complete the survey.

Interestingly, only 72.4% of caregivers identified pathologists as medical doctors before education. However, almost all caregivers (98.3%) were able to identify pathologists as medical doctors after education. While caregivers benefited from pathology report education regardless of educational background, it seems that caregivers with lower educational levels may benefit more from pathology report education. Similarly, caregivers with patients having their first Peds GI procedure may benefit more from pathology report education. This project worked with highly educated caregivers, 54.6% of which had at least a college degree. To further test the conclusion that pathology report education most benefits those with lower educational backgrounds, future studies should involve other sites. These studies should target those with lower educational backgrounds and those with less access to care. Furthermore, one limitation of this study is that the educational materials and survey were only printed in English and only given to caretakers using English as their first language. Future work should, as a start, include Spanish and Arabic materials alongside efforts to include non-English-speaking families. Also, the educational handout used in this project is at a Flesch-Kincaid reading grade level of 14.7. A more accessible version of the handout with a Flesch-Kincaid reading grade level of 9.7 (Supplemental Digital Content File 3, http://links.lww.com/PG9/A82) has been included for future studies. The high reading grade level used in this study could be one reason why caregivers with higher educational backgrounds benefited more from education. Likewise, many of the correct survey answers have the exact verbiage used in the educational handout. This likely biased the correct number of survey responses upwards, perhaps more so for those with higher reading levels.

Future work should also target specific diagnoses. For example, patients potentially receiving a terminal diagnosis via their pathology report may greatly benefit from prior pathology report education. This is especially true with patients now receiving pathology reports at the same time as the ordering physician. Future work should also target populations not using online patient portals. Although these patients and caregivers may not receive their pathology reports directly, they may represent a population with lower health literacy that still would benefit from education regarding their care.

The total number of caregivers offered the survey and educational handout was not tracked. Anecdotally, no >4 to 7 caregivers refused the survey, making the response rate about 90%. Studies (8) have examined patient satisfaction with and patient preferences for waiting and holding areas, but studies administering surveys in the holding or waiting area are not readily findable. The preoperative period could be a new opportunity for survey administration.

This project has many additional limitations. The survey and handout only questioned specific aspects of pathology and pathology reports. In addition, caregivers were not contacted after receiving the pathology report to learn if education was helpful. Furthermore, pathologists were not consulted to discuss adding education to pathology reports themselves. Pathology reports could include simple explanations of pathology reports, pathologists’ roles, and histological findings. Current literature explores adding education to pathology reports and creating web-based tools for pathology education (9,10).

Despite its limitations, the context underlying this project is applicable to medical centers across the United States. The 21st Century Cures Act is making patient data immediately available. These data include pathology reports, for which there is little patient education. Different medical centers are at different stages of incorporating the 21st Century Cures Act. However, all pathology reports at medical centers with electronic medical records and patient portals will eventually become available to patients. Thus, while the results of this project reflect data from only one site, the importance of pathology patient education is generalizable to many medical centers. The results of this project are also unique. Previously, the efficacy of an educational handout for pathology reports had not been assessed with a pre- and postsurvey. In addition, specific target populations for pathology education had not been identified with statistical measures.

Traditionally, pathology reports convey the pathologist’s findings to the ordering physician, creating no reason for patient education to exist for pathology reports. Now, patients and their families should be considered as a new target audience for pathology reports. Education of physicians, caregivers, and their families should be developed accordingly and deployed before patients receive their pathology reports. In anticipation of the 21st Century Cures Act’s effects, pathology report education will have a positive impact on patients, their caregivers, and their physicians. An attainable goal would be for every patient or caregiver to receive education before their pathology report becoming available on the online patient portal. This education could come from the surgeon or from the pathologist. Education could start now with telling the patient or caregiver preoperatively they will receive a pathology report online.

## Supplementary Material


