# Supplementary material for: Education for Pediatric Gastroenterology Pathology Reports Increases Understanding Ahead of 21st Century Cures Act Rollout
Source: JPGN Rep. 2022 Mar 31;3(2):e197. doi: 10.1097/PG9.0000000000000197 (PMC10158402; doi:10.1097/PG9.0000000000000197)
Supplement: Supplementary file 1 [file pg9-3-e197-s001.pdf]

Pediatric Gastroenterology Quality Improvement Survey

This is a Quality Improvement project. We hope to understand areas for improvement in the MyHealthAtVanderbilt patient portal. We want to increase patient/caregiver understanding and satisfaction when viewing pediatric gastroenterology results in the portal.

Will the patient or a caregiver be taking this survey?
- ◯ Patient
- ◯ Family member

If the patient is taking the survey - how old is the patient?
____

If a caregiver, not the patient, is taking the survey - what is the highest education level of the family member taking the survey?
- ◯ Some high school
- ◯ High school degree
- ◯ Some college
- ◯ College degree
- ◯ Graduate/Professional degree

Does the patient have a MyHealthAtVanderbilt account?

MyHealthAtVanderbilt is our online patient portal. If NO, please do not complete this survey.
- ◯ Yes
- ◯ No

This is a Quality Improvement project. We hope to understand areas for improvement in the MyHealthAtVanderbilt patient portal. We want to increase patient/caregiver understanding and satisfaction when viewing pediatric gastroenterology results in the portal.

Is this the patient's first pediatric gastroenterology procedure? (Colonoscopy, endoscopy, etc)	◯ Yes<br>◯ No
What procedure is the patient having today?	◯ Upper scope (EGD or esophagogastroduodenoscopy)<br>◯ Lower scope (colonoscopy)<br>◯ Upper and lower scope (EGD + colonoscopy)<br>◯ Upper scope (EGD) and flexible sigmoidoscopy<br>◯ Other (please write below)<br>(If "Other", please write the procedure below:)

This is a Quality Improvement project. We hope to understand areas for improvement in the MyHealthAtVanderbilt patient portal. We want to increase patient/caregiver understanding and satisfaction when viewing pediatric gastroenterology results in the portal.

After their upcoming pediatric gastroenterology procedure, the patient will receive a PATHOLOGY REPORT on MyHealthAtVanderbilt.

What does a pathology report contain?

- ◯ Images collected during the procedure
- ◯ Information about the quality of the doctor who performed the procedure
- ◯ Analysis of tissue collected during the procedure
- ◯ None of the above
- ◯ Unsure

Please rate your agreement with the following statement:

I know what sorts of information to expect from the pathology report the patient will receive after their pediatric gastroenterology procedure.

- ◯ Strongly disagree
- ◯ Disagree
- ◯ Neutral
- ◯ Agree
- ◯ Strongly agree

Who will write the pathology report created after the patient's pediatric gastroenterology procedure?

- ◯ A pathologist
- ◯ The physician who performed the procedure

This is a Quality Improvement project. We hope to understand areas for improvement in the MyHealthAtVanderbilt patient portal. We want to increase patient/caregiver understanding and satisfaction when viewing pediatric gastroenterology results in the portal.

Are pathologists practicing physicians?

Practicing physicians graduated from medical school and then did additional training in their field of interest.

- ◯ Yes, pathologists are practicing physicians
- ◯ No, pathologists are NOT practicing physicians

What topics do you wish you received patient education about?

____

Are you satisfied with the MyHealthAtVanderbilt patient portal?

- ◯ Yes
- ◯ No

Do you have any suggestions to improve the MyHealthAtVanderbilt patient portal?

____

PLEASE READ THE EDUCATIONAL HANDOUT BEFORE PROCEEDING

When you've read the educational handout, mark the button: ◯ I've read the educational handout

This is a Quality Improvement project. We hope to understand areas for improvement in the MyHealthAtVanderbilt patient portal. We want to increase patient/caregiver understanding and satisfaction when viewing pediatric gastroenterology results in the portal.

Are pathologists practicing physicians?<br><br>Practicing physicians graduated from medical school and then did additional training in their field of interest.	◯ Yes, pathologists are practicing physicians<br>◯ No, pathologists are NOT practicing physicians
After their upcoming pediatric gastroenterology procedure, the patient will receive a PATHOLOGY REPORT on MyHealthAtVanderbilt.<br><br>What does a pathology report contain?	◯ Images collected during the procedure<br>◯ Information about the quality of the doctor who performed the procedure<br>◯ Analysis of tissue collected during the procedure<br>◯ None of the above<br>◯ Unsure
Please rate your agreement with the following statement:<br><br>I know what sorts of information to expect from the pathology report the patient will receive after their pediatric gastroenterology procedure.	◯ Strongly disagree<br>◯ Disagree<br>◯ Neutral<br>◯ Agree<br>◯ Strongly agree
Who will write the pathology report created after the patient's pediatric gastroenterology procedure?	◯ A pathologist<br>◯ The physician who performed the procedure
