# Supplementary material for: Education for Pediatric Gastroenterology Pathology Reports Increases Understanding Ahead of 21st Century Cures Act Rollout
Source: JPGN Rep. 2022 Mar 31;3(2):e197. doi: 10.1097/PG9.0000000000000197 (PMC10158402; doi:10.1097/PG9.0000000000000197)

Patient Educational Handout: Pediatric Gastroenterology Pathology Reports

- After having a procedure done by a pediatric gastroenterology doctor, the patient will receive a **pathology report** on the MyHealthAtVanderbilt portal.
- A **pathology report** is a microscopic analysis of tissue samples taken during the procedure. This report explains what the cells from that tissue sample look like.
- Pathology reports are created by **pathologists**, who are practicing medical doctors (a.k.a. physicians) that have received specialized pathology training.
- Pathology report results help pediatric gastroenterology doctors explain the outcome of the procedure. **Some findings on pathology reports are not clinically significant. The final interpretation will be made by the pediatric gastroenterologist once they review the report.**
- So that you know what to expect – here are some **examples of normal pathology report results** for different pediatric gastroenterology procedures:

Normal EGD Pathology Report

Diagnosis

1) DUODENUM, BIOPSY: DUODENAL MUCOSA WITH NORMAL VILLOUS ARCHITECTURE; NO SIGNIFICANT HISTOPATHOLOGIC CHANGE.

2) DUODENAL BULB, BIOPSY: DUODENAL MUCOSA WITH NORMAL VILLOUS ARCHITECTURE; NO SIGNIFICANT HISTOPATHOLOGIC CHANGE.

3) STOMACH, BIOPSY: NO SIGNIFICANT HISTOPATHOLOGIC CHANGE. NEGATIVE FOR HELICOBACTER LIKE ORGANISMS.

4) ESOPHAGUS, DISTAL, BIOPSY: NO SIGNIFICANT HISTOPATHOLOGIC CHANGE. NEGATIVE FOR INTRAEPITHELIAL EOSINOPHILS.

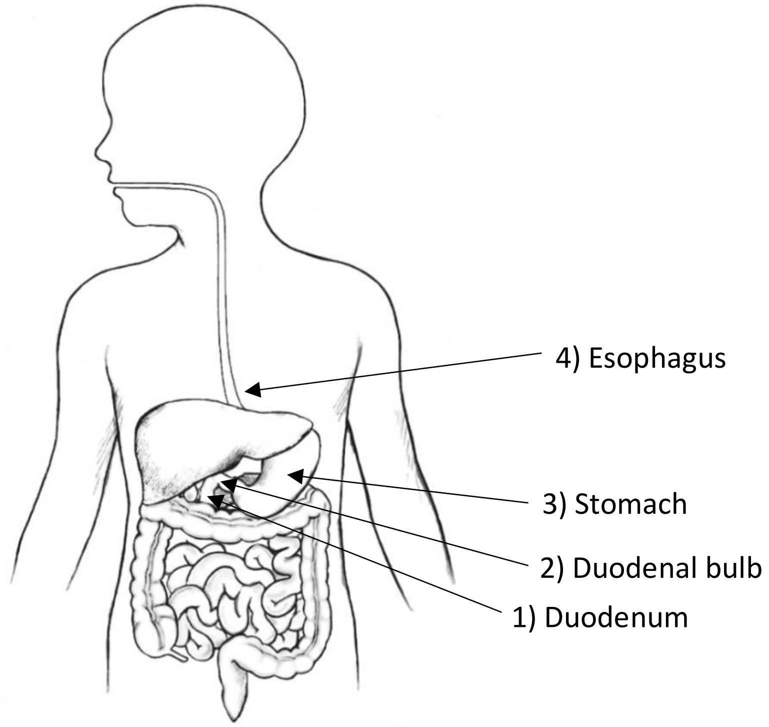

Normal EGD+Colonoscopy Pathology Report

Diagnosis

1) DUODENUM, BIOPSIES:
   - DUODENAL MUCOSA WITH NO DIAGNOSTIC ALTERATIONS.
   - NEGATIVE FOR INCREASED INTRAEPITHELIAL LYMPHOCYTES AND VILLOUS BLUNTING IN EVALUABLE AREAS.

2) STOMACH, BIOPSIES:
   - ANTRAL AND OXYNTIC MUCOSA WITH NO DIAGNOSTIC ALTERATIONS
   - NO HELICOBACTER PYLORI ORGANISMS IDENTIFIED ON H&E STAIN.

3) DISTAL ESOPHAGUS, BIOPSIES:
   - SQUAMOUS EPITHELIUM WITH NO DIAGNOSTIC ALTERATIONS
   - NEGATIVE FOR INCREASED INTRAEPITHELIAL EOSINOPHILS.

4) TERMINAL ILEUM, BIOPSIES:
   - ILEAL MUCOSA WITH NO DIAGNOSTIC ALTERATIONS.

5) RIGHT/ASCENDING COLON, BIOPSIES:
   - COLONIC MUCOSA WITH NO DIAGNOSTIC ALTERATIONS.

6) LEFT/DESCENDING COLON, BIOPSIES:
   - COLONIC MUCOSA WITH NO DIAGNOSTIC ALTERATIONS.

7) RECTUM, BIOPSIES:
   - COLONIC MUCOSA WITH NO DIAGNOSTIC ALTERATIONS.

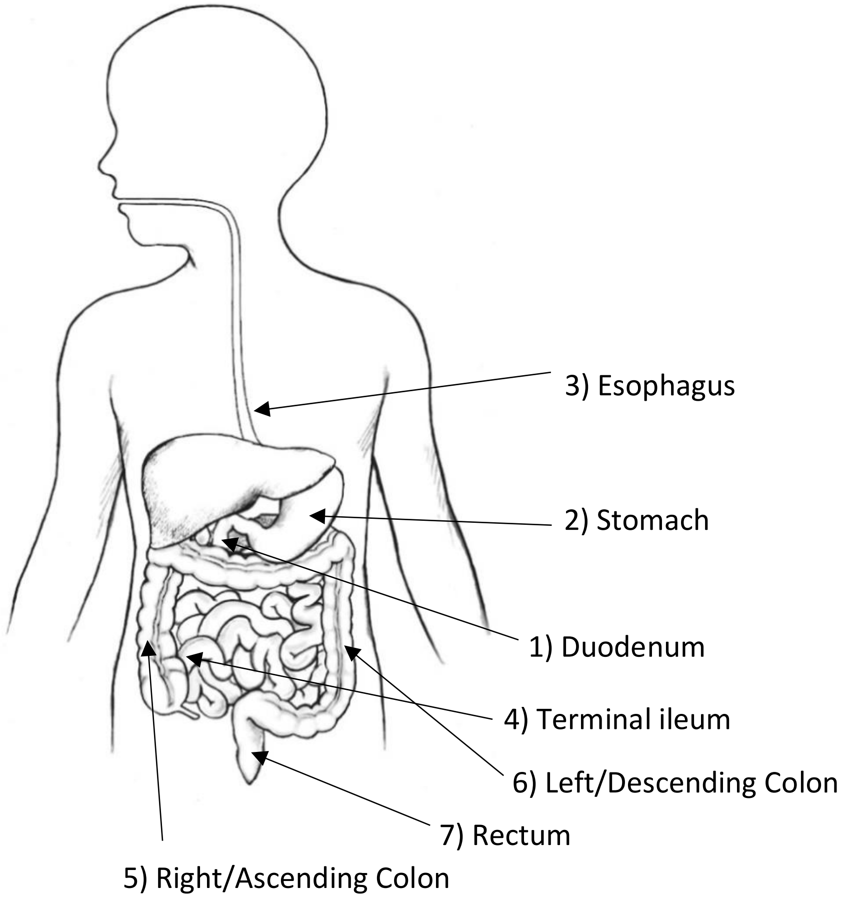

Supplement: Supplementary file 2 [file pg9-3-e197-s002.pdf]
